# Lifetime and childhood traumatic experiences as risk factors for persistent post-concussive symptoms among US service members

**DOI:** 10.3389/fneur.2026.1816906

**Published:** 2026-05-28

**Authors:** Rosemay A. Remigio-Baker, Jan Kennedy, Lisa Lu, Matthew W. Reid, Jessica Bock, Michael Shaughness, Alexis Nelson, Mira R. Ananthanarayanan, Jamie Hershaw

**Affiliations:** 1 Traumatic Brain Injury Center of Excellence (TBICoE), Silver Spring, MD, United States; 2 Compass Government Solutions, Annapolis, MD, United States; 3 Joint Base San Antonio, San Antonio, TX, United States; 4 General Dynamics Information Technology, Falls Church, VA, United States; 5 CICONIX, Annapolis, MD, United States; 6 Naval Hospital Camp Pendleton, Camp Pendleton, CA, United States

**Keywords:** childhood traumatic experiences, concussion, lifetime traumatic experiences, mental health, mild traumatic brain injury, military, persistent post-concussive symptoms

## Abstract

**Objectives:**

The objectives of this study were to investigate how the number of lifetime and childhood traumatic experiences relates to persistent post-concussive symptoms (PPCS) and mental health symptoms among US service members (SMs).

**Methods:**

This study included 65 SMs who belonged to one of three groups: (1) 18 with mild traumatic brain injury (mTBI) and *no* PPCS; (2) 18 with mTBI and PPCS; and (3) 29 with an extra-cranial injury but no mTBI. Lifetime traumatic experiences were measured using the Trauma History Screen (THS) and childhood traumatic experiences were measured using the Adverse Childhood Experiences (ACEs) survey. The outcomes included severity of post-traumatic stress disorder (PTSD) measured by Clinician Administered PTSD Scale-5, the level of post-traumatic stress symptoms measured by the PTSD Checklist, DSM-5, and the level of mental health symptoms measured by the Brief Symptom Inventory.

**Results:**

A greater number of lifetime (but not childhood) traumatic experiences were found among those with mTBI and PPCS compared to those with mTBI but *no* PPCS or no mTBI; however a greater number of childhood traumatic experiences were associated with poor mental health symptoms in areas of somatization and interpersonal sensitivity, but only among those with mTBI and PPCS.

**Conclusions:**

The findings of this exploratory study show that the number of lifetime traumatic experiences may be related to persistent post-concussive symptoms; however, only the number of childhood trauma was associated with mental health symptoms among those with persistent symptoms. These findings support screening for childhood and lifetime traumatic experiences during evaluations at clinic intake to improve risk assessment, treatment planning, and recovery expectations and outcomes. While the results may highlight the need for early identification of at-risk individuals for persistent symptoms to enhance readiness and return-to-duty rates, further studies are needed to elucidate the mechanisms of these associations.

## 1 Introduction

Deployment to combat zones such as the Operation Iraqi Freedom (OIF) and Operation Enduring Freedom (OEF) has contributed to an increased prevalence of traumatic brain injury (TBI) among US service members (SMs). According to the Traumatic Brain Injury Center of Excellence, over 528,000 SMs have sustained a TBI since the year 2000, 82% of which are mild in severity (i.e., concussion) (1). Mild TBI (mTBI) is associated with a multitude of post-injury symptoms encompassing poor cognitive, physical and mental health. Although the vast majority of SMs who sustain mTBI fully recover from post-concussive symptoms within 3 months, about 10–15% experience persistent symptoms known as persistent post-concussive symptoms (PPCS) (2). Recent research has shown an association between mental health outcomes and PPCS where individuals with PCSS are at higher risk for mental health issues (3). For example, in a scoping review of mental health outcomes across a lifetime among individuals with mTBI, most studies reported greater anxiety and depressive symptomatology in those with PPCS compared to those who have recovered or were healthy controls (4). This body of literature suggest that, if mental health symptoms remain untreated, they may, in part, contribute to the maintenance of PPCS. Compared to the civilian population, SMs are at a greater risk for sustaining mTBIs due to exposure to high kinetic events in theater and training (e.g., blast overpressure exposure, combatives and heavy weapons training) and poorer mental health outcomes because of unique stressors (e.g., combat exposure and prolonged deployment) (5). Therefore, it is crucial to identify contributing factors that may maintain poor mental health in this population, and may serve as targets for interventions aimed at improving mTBI treatment and facilitating recovery and return to duty.

Greater levels of pre-injury psychosocial adversity and/or family dysfunction have been shown to predict greater affective lability and more clinically significant behavioral problems following mTBI (6). Additionally, studies have shown that lifetime traumatic events augment poor mental health and lower quality of life (7–9). Such experiences can exacerbate current health ailments among individuals with mTBI or contribute to the persistence of chronic conditions (e.g., pain, cognitive dysfunction) (10). Adverse childhood experiences (ACEs) which include household dysfunction, physical, emotional and sexual abuse, and neglect before the age of 18, have also been shown to lead to negative psychological health in adulthood (7, 8, 11, 12). Specifically, a history of childhood maltreatment among women has been shown related to a greater likelihood for emotional difficulties that impair daily functioning and lifetime psychopathology such as anxiety, alcohol abuse/dependence and depression (7, 8, 11, 12). Additionally, a greater impact of childhood abuse on mental health has been shown among those with co-existing conditions, such as cardiovascular disease, compared to the general population (13). As such, mTBI in the presence of lifetime or childhood traumatic experiences may negatively impact the level of mental health symptoms in an additive or multiplicative manner. In turn, elevated mental health symptoms may promote the persistence of post-concussive symptoms. Although there are different types of trauma that individuals encounter during a lifetime, an increasing number of these exposures are also known to contribute to worse health outcomes (14). For example, a dose response has been demonstrated between the number of ACEs and the odds of current depressive symptoms (11). Understanding this complex relationship may further advance knowledge of recovery trajectory from mTBI.

While lifetime and childhood traumatic events are shown associated with poor mental health outcomes (11, 15–17), their role in PPCS remains understudied. Adversity is likely not the sole factor contributing to persistent symptoms following mTBI; however, a plethora of evidence supports the association between early adversity and increased morbidity and mortality across a host of diseases and disorders, including the increasing prevalence of elevated mental health symptoms (11, 18, 19). As such, psychosocial adversity and family dysfunction may contribute to PPCS and impact military operational readiness and recovery (20, 21).

The objectives of this study are to determine if the number of lifetime and childhood traumatic experiences is related to the presence or absence of PPCS, and whether the association between traumatic experiences and psychological problems differ based on the persistence of post-concussive symptoms.

## 2 Materials and methods

Data for this cross-sectional study was obtained from a 3-cohort pilot study investigating the impact of adversity on post-concussive symptom endorsement in SMs who sustained mTBI. Patients were recruited for the pilot study from individuals responding to written advertisement, through Soldier Readiness Program events, and referrals from the Brooke Army Medical Center neurology, brain injury and rehabilitation, and emergency departments. Recruitment flyers were also posted throughout the hospital to attract participants. Interested individuals were contacted by a researcher who explained the study procedures and answered questions regarding the study. A brief interview was completed to ensure study eligibility. Eligible participants who agreed to be in the study provided written consent. The study protocol was approved by the San Antonio Institutional Review Board.

### 2.1 Participants

This study included 65 SMs from a pilot study based on the following categorization: 18 with a history of mTBI with PPCS; 18 with a history of mTBI with no PPCS; and 29 controls with no history of mTBI. Classification of mTBI was based on a loss of consciousness lasting no more than 30 min; alteration of consciousness lasting at least 5 min, but not more than 24 h; and post-traumatic amnesia lasting no more than 24 h post-injury (22). Participants with mTBI and PPCS were those who sustained a deployment-related mTBI, were symptomatic and were seeking treatment. Those with mTBI and *no* PPCS were SMs who sustained mTBI but did not report mTBI symptoms and had not sought or received treatment for mTBI. Those with no mTBI were SMs who sustained an extracranial injury with no history of TBI. All participants were 18–65 years of age; had a post-injury interval of > 2 months; and performed above cut-off scores on the Word Memory Task in accordance with the manual. Participants were excluded if they were not fluent in English; they had pre-existing neurologic disorder associated with cerebral dysfunction and/or cognitive deficit; were diagnosed with learning disabilities or dyslexia; had pre-existing severe psychiatric disorder; had post-deployment TBI with hospitalization; had burn injury over more than 10% of their body area; or were unwilling to participate in the study.

### 2.2 Measures

#### 2.2.1 Clinician-administered PTSD Scale for DSM-5

The severity of PTSD was measured using the 20-item Clinician-Administered PTSD Scale for DSM-5 (CAPS-5) to evaluate symptoms ranging from absent to extremely decapacitating, which have occurred in the past week (23). This gold standard was administered by clinical researchers with a working knowledge of PTSD. A single severity rating for each item was calculated by combining information about the frequency and intensity of said item. A summation of the severity score for the 20 DSM-5-defined PTSD symptoms was then calculated into a CAPS-5 total symptom severity score. CAPS-5 has demonstrated strong interrater (*K* = 0.78–1.00) and test-retest reliability (*K* = 0.83), as well as high internal consistency (Cronbach's alpha = 0.88) and interrater reliability (ICC = 0.91), and good test-retest reliability (ICC = 0.78) (24). It also shows good convergent validity with the PTSD Checklist for DSM-5 (*r* = 0.66) and good discriminant validity with measures of psychopathy, disability and alcohol abuse (*r* = 0.02–0.54) (24).

#### 2.2.2 PTSD Checklist for DSM-5

Assessment of self-reported post-traumatic symptoms (PTS) used the PTSD Checklist for DSM-5 (PCL-5), a 20-item survey that evaluates the extent to which patients were bothered by symptoms of PTS in the past month (25). Each of the responses was based on a Likert scale ranging from 0 (“not at all”) to 4 (“extremely”), and a summary score was derived from all the items. The PCL-5 has strong test-retest reliability (*r* = 0.82), internal consistency (Cronbach's alpha = 0.94), discriminant validity (rs = 0.31–0.60), and convergent validity (rs = 0.74–0.85) (26).

#### 2.2.3 Brief Symptom Inventory

A wide range of psychological symptoms was measured using the 53-item Brief Symptom Inventory (BSI). This included 9 symptom dimensions: somatization, obsession-compulsion, interpersonal sensitivity, depression, anxiety, hostility, phobic anxiety, paranoid ideation, and psychoticism (27). Responses indicating the extent to which participants were bothered by these symptoms (0 = “not at all” to 4 = “extremely”) were summarized for all items within each dimension and divided by the total number of items within each dimension to produce an average score (27). Three global indices were also measured: the Global Severity Index (GSI, mean score of all BSI items); the Positive Symptom Total (PST, count of all non-zero responses); and the Positive Symptom Distress Index (PSDI, sum of all BSI items with non-zero responses divided by the PST) (27). All raw scores were converted to *T*-scores based on the BSI manual. The BSI is a self-administered survey that rates the severity of various symptoms experienced in the past week (27). The BSI has been validated against other mental health measures showing correlations ranging from 0.30 to 0.72 when assessed against the Wiggins content scales and the Tryon cluster scores from the Minnesota Multiphasic Personality Inventory (28). Correlations between BSI and Symptom Checklist-90 ranged from 0.92 to 0.99 (29). The BSI also shows good internal consistency for the nine dimensions from 0.71 (psychoticism) to 0.85 (depression) (29). No Cronbach's alpha is reported for the 3 global indices. Test-retest reliability shows a range from 0.68 (somatization) to 0.91 (phobic anxiety) for the 9 dimensions, and from 0.87 (PSDI) to 0.90 (GSI) for the global indices (29).

#### 2.2.4 Trauma History Screen

Lifetime trauma was assessed using the 14-item Trauma History Screen (THS) which was developed as a quick evaluation of exposure to a broad range of high magnitude stressors (i.e., sudden events that cause extreme distress for most), traumatic stress (i.e., events that cause extreme distress for an individual) and persisting post-traumatic distress (i.e., events linked to significant subjective distress that lasts for more than a month) (30). Participants were asked if they experienced traumatic events such as physical (e.g., hit/kicked as a child/adult) and sexual (e.g., forced or made to have sexual contact as a child/adult) abuse, seeing someone die/get hurt/killed, natural disasters (e.g., hurricane, flood, earthquake, tornado, or fire), accidents (e.g., a really bad car accident), harassment (e.g., attack with gun or knife or weapon), being scared during military service, loss (e.g., sudden death of close family/friend; sudden move or loss of home and possessions), and/or getting suddenly abandoned by spouse, partner, parent, or friend. Responses were either yes or no. The age when these events happened and the number of times they occurred were also captured using this screener. A summation of the occurrence of any traumatic events was used in analyses. Only one participant reported no history of lifetime trauma and, thus, this variable was not evaluated for presence vs. absence. The test-retest reliability of THS has been shown to be relatively strong, ranging from 0.73 to 0.95 (30). It also shows good convergent validity showing strong correlation between reported military events and exposure to military stressors on the Combat Exposure Survey (*r* = 0.57 to 0.81) (30).

#### 2.2.5 Adverse Childhood Experiences

History of childhood trauma was measured using the 10-item Adverse Childhood Experiences (ACEs) survey which included questions regarding household dysfunction, physical, emotional and sexual abuse, and neglect that occurred prior to 18 years of age (9). For example, participants were asked, “Did you live with a problem drinker/street drug use,” “Did a parent/adult push or hit you,” “Did a parent/adult put you down or make you afraid,” “Did a parent/older person ever touch you,” and “Did you often feel you didn't have enough to eat.” Responses were either yes or no. A summation of the total number of ACE items with “yes” responses was used for analyses. Given the limited sample size, evaluation by the type of ACEs (e.g., physical abuse, neglect, etc.) was not conducted. The ACE survey originated from the Center for Disease Control and Prevention-Kaiser Permanente ACE Study and has shown good reliability (ω = 0.906) and structural validation properties, strong test-retest reliability (*r* = 0.913) as well as adequate internal consistency (Cronbach's alpha ranging from 0.75 to 0.79) (31–34).

### 2.3 Statistical analysis

The demographic and military characteristics of the study sample were summarized in total and stratified by group (i.e., no mTBI, mTBI with *no* PPCS, and mTBI with PPCS). Due to the limited cell sample sizes across mTBI groups, adjustments for covariates or assessment for potential modifiers were not conducted. Bootstrapped statistical approaches were, however, utilized to address the limited sample size. To compare the number of lifetime and childhood traumatic experiences across the three mTBI groups, a bootstrapping method with 1,000 repetitions was conducted to obtain Hedge's g estimation and a 95% confidence interval to determine statistical significance. Bootstrap Spearman's rank correlation coefficient was obtained with 1,000 repetitions to evaluate the correlation between the number of lifetime and childhood traumatic events and the level of mental health symptoms, overall and stratified by mTBI group. All analyses were conducted using the Stata software v.15 (StataCorp 2017, College Station, TX; RRID: SCR_012763).

## 3 Results

Table 1 summarizes the study sample characteristics overall and by the three mTBI groups. The sample had a mean age of 38.7 years (*SD* = 7.5) and were mainly males (81.8%) and non-Hispanic White (65.2%). Almost half had at least a 4-year college degree (45.5%), and most were married (84.9%), served in the Army Branch (78.5%) and were active duty (78.8%). When stratified by mTBI group, those with mTBI and *no* PPCS had a greater proportion of males; those with no mTBI had more non-Hispanic White; those with mTBI and PPCS were less likely to have at least a 4-year college degree; and those with mTBI and PPCS had a greater proportion serving in the Army compared to the other groups. However, caution must be taken in interpreting these differences as sample size was limited, with some categories having zero participants within each group.

**Table 1:** Characteristics of study sample, overall and by mTBI group.

Characteristic	All *N* = 65	mTBI group		
		mTBI and PPCS *n* = 18	mTBI and *no* PPCS *n* = 18	No mTBI *n* = 29
Age in years				
Mean (SD)	38.7 (7.6)	39.9 (7.3)	38.7 (7.5)	38.0 (7.9)
Median (IQR)	38 (34, 44)	39 (35, 45)	40 (35–45)	37 (32, 42)
Range	25–58	29–52	25–52	27–58
Sex, *n* (%)				
Female	12 (18.5)	0 (0)	2 (11.1)	10 (34.5)
Male	53 (81.5)	18 (100)	16 (88.9)	19 (65.5)
Race and ethnic group, *n* (%)				
Hispanic	12 (18.5)	6 (33.3)	5 (27.8)	1 (3.5)
NH black	6 (9.2)	0 (0)	4 (22.2)	2 (6.9)
NH other	5 (7.7)	2 (11.1)	1 (5.6)	2 (6.9)
NH white	42 (64.6)	10 (55.6)	8 (44.4)	24 (82.8)
Education, *n* (%)				
High school diploma/GED	14 (21.5)	5 (27.8)	5 (27.8)	4 (13.8)
Business/tech/trade/certificate	4 (6.2)	3 (16.7)	0 (0)	1 (3.5)
Associate degree	16 (24.6)	5 (27.8)	3 (16.7)	8 (27.6)
Bachelor's degree	5 (7.7)	2 (11.1)	2 (11.1)	1 (3.5)
Master degree	19 (29.2)	2 (11.1)	8 (44.4)	9 (31.0)
Doctoral degree	6 (9.2)	0 (0)	0 (0)	6 (20.7)
Other	1 (1.5)	1 (5.6)	0 (0)	0 (0)
Marital status, *n* (%)				
Single/never married	4 (6.2)	0 (0)	1 (5.6)	3 (10.3)
Married	55 (84.6)	15 (83.3)	15 (83.3)	25 (86.2)
Divorced	6 (9.2)	3 (16.7)	2 (11.1)	1 (3.5)
Service branch, *n* (%) (*n* = 49)				
Army	37 (75.5)	17 (94.4)	2 (100)	18 (62.1)
Air force	11 (22.5)	0 (0)	0 (0)	11 (37.9)
Navy	1 (2.0)	1 (5.6)	0 (0)	0 (0)
Military status, *n* (%)				
Active duty	51 (78.5)	14 (77.8)	16 (88.9)	22 (73.3)
Reserves	4 (6.2)	1 (5.6)	1 (5.6)	2 (6.7)
Retired	7 (10.8)	1 (5.6)	1 (5.6)	5 (16.7)
Honorably discharged	3 (4.6)	2 (11.1)	0 (0)	1 (3.3)

mTBI, mild traumatic brain injury; PPCS, persistent post-concussive symptoms: NH, non-Hispanic.

### 3.1 Frequency of lifetime and childhood traumatic experiences across mTBI groups

Considering all participants regardless of mTBI status, nearly all participants had at least one lifetime traumatic experience. By mTBI status, those with mTBI and PPCS had a median of 7.5 lifetime traumatic experiences (IQR = 6, 10; range = 4–12). Those with mTBI and *no* PPCS had a median of 5 lifetime traumatic experiences (IQR = 4, 6; range = 1–9), and those with no mTBI history had a median of 6 lifetime traumatic experiences (IQR = 4, 7; range = 0–10).

Approximately 86% of participants had at least one ACE. All of those with mTBI and PPCS had at least one ACE and, among them, there was a median of one ACEs (IQR = 1, 2; range = 1–5). Of those with mTBI and *no* PPCS, 74% had at least one ACE and, among them, there was a median of two ACEs (IQR = 0, 4; range = 0–6). Among SMs with no mTBI, about 92% had at least one ACE, and, among them, there was a median of two ACEs (IQR = 1, 4; range = 0–10).

Table 2 shows the results comparing the number of lifetime or childhood traumatic experiences across mTBI groups. Statistical significance and a large effect size were only found in the comparison between the mTBI and PPCS and mTBI and *no* PPCS groups, whereby participants with mTBI and PPCS were significantly more likely to report a greater number of lifetime traumatic experiences compared to those with mTBI and *no* PPCS. However, moderate effect size, though non-significant, was found in the comparisons between either mTBI and PPCS or mTBI and *no* PPCS vs. no mTBI for lifetime traumatic experiences, and between mTBI and PPCS and no mTBI for childhood traumatic experiences (i.e., effect size >0.5).

**Table 2:** Group comparisons of the number of lifetime or childhood traumatic events (*n* = 65).

Trauma	mTBI group comparisons Hedges' g (95% ***CI***)^**a, b**^		
	mTBI with PPCS vs. mTBI and *no* PPCS (ref)	mTBI with PPCS vs. No mTBI (ref)	mTBI and *no* PPCS vs. No mTBI (ref)
Number of lifetime traumatic events	**1.18 (0.38, 1.98)** ^*^	0.77 (−0.04, 1.58)	−0.52 (−1.24, 0.20)
Number of childhood traumatic events	−0.21 (−0.93, 0.52)	−0.60 (−1.32, 0.13)	−0.47 (−1.24, 0.29)

mTBI, mild traumatic brain injury; PPCS, persistent post-concussive symptoms; ref, reference.

^a^Utilized bootstrapping method to obtain Hedge's *g* estimation and a 95% confidence interval.

^b^Hedges' *g* statistic expresses the difference of the means in units of the pooled standard deviation. Its interpretation is as follows:

•**Small effect size** (difference may be noticeable but not substantial): between 0.2 and < 0.5. •**Medium effect size** (indicating a moderate difference): Around 0.5. •**Large effect size** (suggesting substantial and noticeable difference): > 0.8.

^*^Significant *p*-value < 0.05 (bolded).

### 3.2 Frequency of lifetime and childhood traumatic experiences and level of mental health symptoms across mTBI groups

Table 3 summarizes the results evaluating the relationship between the number of lifetime and childhood traumatic experiences and the level of mental health symptoms overall. A greater number of lifetime traumatic experiences was significantly correlated with greater PTSD severity and PTS symptom level. There was also a positive correlation between the number of lifetime traumatic experiences and somatization. There was no significant correlation between the number of childhood traumatic experiences and any mental health symptoms assessed; however, significances appeared when stratified by mTBI groups. Table 4 shows the results when determining whether the correlation between the number of lifetime or childhood traumatic experiences and mental health symptoms differ across mTBI groups. A significant positive correlation was found between lifetime traumatic experiences and somatization, but only among those with mTBI and *no* PPCS. Among those with no mTBI, a greater number of lifetime traumatic experiences was correlated with a higher level of interpersonal sensitivity. When considering the number of childhood traumatic experiences, significance was found among those with mTBI and PPCS whereby those with a greater number of childhood traumatic experiences were more likely to have a greater level of somatization and lower levels of interpersonal sensitivity. Among those with no history of mTBI, those with a greater number of lifetime or childhood traumatic experiences had greater levels of PTS symptoms.

**Table 3:** Correlation between number of lifetime and childhood traumatic events and level of mental health symptomatology, overall (*n* = 65).

Level of mental health symptoms	Bootstrap Spearman Rank Correlation Coefficient (95% Confidence Interval)	
	Number of lifetime traumatic events	Number of childhood traumatic events
Clinician-administered PTSD scale for DSM-5 (CAPS-5)		
15.6-7.4,-1.3242ptTotal severity	**0.44 (0.15, 0.74)** ^*^	0.15 (−0.16, 0.47)
PTSD Checklist, DSM-5		
15.6-7.4,-14.3242ptPost-traumatic stress symptoms as PCL-5 score	**0.40 (0.08, 0.73)** ^*^	0.13 (−0.18, 0.43)
Brief symptom inventory as *t*-scores		
Somatization	**0.38 (0.09, 0.67)** ^*^	−0.15 (−0.48, 0.17)
Obsession-compulsion	0.18 (−0.15, 0.51)	0.08 (−0.24, 0.40)
Interpersonal sensitivity	0.23 (−0.07, 0.53)	−0.13 (−0.46, 0.21)
Depression	0.18 (−0.13, 0.49)	−0.002 (−0.37, 0.37)
Anxiety	0.21 (−0.12, 0.54)	0.09 (−0.26, 0.44)
Hostility	0.16 (−0.14, 0.46)	0.03 (−0.29, 0.35)
Phobic anxiety	0.23 (−0.09, 0.54)	−0.14 (−0.46, 0.18)
Paranoid ideation	0.13 (−0.21, 0.46)	−0.02 (−0.33, 0.29)
Psychoticism	0.13 (−0.18, 0.44)	−0.13 (−0.47, 0.20)
Global severity index	0.26 (−0.06, 0.57)	0.03 (−0.31, 0.38)
Positive symptom total	0.30 (−0.0005, 0.61)	−0.01 (−0.34, 0.33)
Positive symptom distress	0.10 (−0.22, 0.42)	0.10 (−0.24, 0.45)

PTSD, post-traumatic stress disorder; PCL-5, PTSD checklist, DSM-5.

Analytic method: Bootstrap Spearman's rank correlation, 1,000 repetitions.

^*^Significant *p*-value < 0.05 (bolded).

**Table 4:** Association between number of lifetime and childhood traumatic events and level of mental health symptomatology among service members with mild traumatic brain injury, by mTBI groups (*n* = 65).

Level of mental health symptoms	Bootstrap Spearman Rank Correlation Coefficient (95% Confidence Interval)					
	Number of lifetime traumatic events			Number of childhood traumatic events		
	mTBI with PPCS	mTBI with *no* PPCS	No mTBI	mTBI with PPCS	mTBI with *no* PPCS	No mTBI
Clinician-administered PTSD Scale for DSM-5 (CAPS-5)						
15.6-5.0,-1.4498.9ptTotal severity	0.21 (−0.55, 0.98)	0.47 (−0.02, 0.95)	0.24 (−0.37, 0.85)	−0.01 (−0.73, 0.72)	0.19 (−0.30, 0.68)	0.48 (−0.09, 1.05)
PTSD Checklist, DSM-5						
15.6-5.0,-26.4498.9ptPost-traumatic stress symptoms, PCL-5 score	0.15 (−0.60, 0.90)	0.37 (−0.17, 0.91)	**0.50 (0.01, 0.996)** ^*^	−0.04 (−0.75, 0.67)	0.15 (−0.32, 0.61)	**0.66 (0.25, 1.07)** ^*^
Brief Symptom Inventory, *t*-scores						
Somatization	0.15 (−0.48, 0.78)	**0.44 (0.01, 0.87)** ^*^	0.30 (−0.32, 0.92)	**0.75 (0.44, 1.07)** ^*^	−0.36 (−0.81, 0.09)	0.04 (−0.59, 0.68)
Obsession-compulsion	0.55 (−0.07, 1.16)	0.27 (−0.22, 0.76)	−0.46 (−0.95, 0.03)	0.19 (−0.61, 0.99)	−0.001 (−0.49, 0.48)	0.50 (−0.0001, 1.01)
Interpersonal sensitivity	0.37 (−0.26, 1.00)	−0.04 (−0.54, 0.47)	**0.40 (0.02, 0.79)** ^*^	–**0.61 (**–**0.99**, –0.23)^*^	−0.05 (−0.54, 0.44)	0.23 (−0.42, 0.89)
Depression	0.02 (−0.65, 0.70)	0.34 (−0.16, 0.83)	0.30 (−0.23, 0.82)	0.19 (−0.50, 0.88)	−0.09 (−0.63, 0.45)	0.16 (−0.52, 0.84)
Anxiety	−0.10 (−0.83, 0.64)	0.09 (−0.45, 0.62)	0.26 (−0.28, 0.81)	0.02 (−0.84, 0.89)	0.06 (−0.42, 0.54)	0.39 (−0.16, 0.93)
Hostility	−0.13 (−0.88, 0.62)	0.10 (−0.37, 0.57)	−0.21 (−0.68, 0.25)	0.40 (−0.27, 1.06)	0.02 (−0.50, 0.54)	0.46 (−0.05, 0.96)
Phobic anxiety	0.34 (−0.40, 1.09)	0.21 (−0.32, 0.76)	0.08 (−0.42, 0.57)	−0.04 (−0.80, 0.72)	−0.26 (−0.73, 0.21)	0.13 (−0.43, 0.70)
Paranoid ideation	0.21 (−0.47, 0.90)	0.10 (−0.45, 0.64)	0.30 (−0.17, 0.77)	0 (−0.75, 0.75)	−0.11 (−0.54, 0.33)	0.39 (−0.18, 0.96)
Psychoticism	0.09 (−0.60, 0.79)	0.19 (−0.33, 0.72)	−0.02 (−0.52, 0.48)	−0.02 (−0.75, 0.70)	−0.20 (−0.70, 0.30)	0.09 (−0.57, 0.76)
Global severity index	0.09 (−0.62, 0.80)	0.22 (−0.26, 0.70)	0.10 (−0.45, 0.65)	0.12 (−0.67, 0.91)	−0.07 (−0.59, 0.46)	0.39 (−0.13, 0.92)
Positive symptom total	0.10 (−0.59, 0.79)	0.28 (−0.23, 0.80)	0.24 (−0.21, 0.70)	0.13 (−0.69, 0.95)	−0.14 (−0.60, 0.32)	0.46 (−0.01, 0.94)
Positive symptom distress symptom	0.42 (−0.18, 1.02)	0.16 (−0.39, 0.71)	−0.19 (−0.74, 0.37)	0.20 (−0.53, 0.94)	0.02 (−0.52, 0.56)	0.29 (−0.24, 0.82)

PPCS, persistent post-concussive symptoms; PTSD, post-traumatic stress disorder; PCL-5, PTSD checklist, DSM-5.

Analytic method: Bootstrap Spearman's rank correlation, 1,000 repetitions.

^*^Significant *p*-value < 0.05 (bolded).

## 4 Discussion

This exploratory study found that the number of lifetime traumatic experiences related to PPCS among participants with mTBI, and that the number of childhood (but not lifetime) traumatic experiences significantly correlated with mental health issues among those with mTBI and PPCS. A moderate effect size was found comparing the number of lifetime traumatic experiences among those with no mTBI vs. either group with mTBI. Specifically, those with mTBI and PPCS had more lifetime traumatic experiences, whereas those with mTBI and *no* PPCS had less lifetime traumatic experiences, compared to participants with no mTBI. A large and significant effect size was found comparing the two groups with mTBI; specifically, those with mTBI and PPCS were more likely to have a greater number of lifetime traumatic experiences compared to those with mTBI and *no* PPCS. Comparing the number of childhood traumatic experiences among participants with no mTBI vs. those with mTBI and PPCS produced a moderate effect size whereby those with mTBI and PPCS had less childhood traumatic experiences. When evaluating the relationship between the number of lifetime or childhood traumatic experiences and mental health symptoms across mTBI groups, a greater number of lifetime traumatic experiences was significantly correlated with higher somatic symptoms among those with mTBI and *no* PPCS and higher PTS symptoms and interpersonal sensitivity among those with no mTBI history. However, a greater number of childhood traumatic experiences were significantly correlated with higher somatic symptoms and lower interpersonal sensitivity among those with mTBI and PPCS. Likewise, a greater number of childhood traumatic experiences among those with no mTBI correlated with higher levels of PTS symptoms. As those with no mTBI had the greatest percentage with at least one ACE and a wider range of number of ACEs, this may reflect the impact of a cumulation of ACEs on PTS symptoms, independent of mTBI.

The results of this exploratory study provide a preliminary understanding of the contribution of lifetime and childhood traumatic experiences on the persistence of post-concussive symptoms among SMs. They extend findings such as that by Meshberg-Cohen et al where among US veterans with mTBI, those with (vs. without) post-concussive symptoms are more likely to have a history of adverse childhood events, cumulative trauma burden and military sexual trauma (35) to the chronicity of these symptoms. The association between lifetime traumatic experiences, particularly during childhood, and poor mental health in adulthood has been well-established. For example, in a prospective, population-based cohort study, the cumulation of childhood trauma was found associated with higher rates of adult psychiatric disorders (15). Other studies have also shown cumulative trauma load to negatively impact life satisfaction, depression and anxiety (16, 17), with childhood trauma, including family dysfunction/neglect and physical, mental and sexual abuse, consistently linked to elevated depressive symptoms in adulthood (11, 36). Based on the World Mental Health survey, 70% of respondents reported at least one lifetime traumatic event, with majority having more than one (37, 38). It is, thus, important to highlight the impact of these traumatic events on conditions such as mTBI and how they may contribute to the persistence of its symptoms as demonstrated in this study. The findings, though preliminary, provide support for the monitoring of lifetime and/or childhood traumatic experiences and administering interventions that target these issues, which may perhaps assist to facilitate and improve recovery from post-concussive symptoms in a timely manner.

Surprisingly, the relationship between lifetime traumatic experiences and mTBI with PPCS was not due to greater levels of mental health symptoms based on the study results. In fact, greater somatization was associated with a greater number of lifetime traumatic experiences, but only among those with mTBI and *no* PPCS. It is possible that somatic symptoms are being addressed through a greater number of clinical visits or therapy for those with mTBI and PPCS. For example, a greater number of visits may increase opportunities for socialization or therapies that help remedy mental health which may decrease somatic symptoms. More so, given the persistence of symptoms, improvement may provide a heightened emotional state which may lessen somatic symptoms. In contrast, although childhood traumatic experiences were not associated with mTBI and PPCS, a greater number of childhood traumatic experiences were associated with a higher level of somatic symptoms among those with mTBI and PPCS. This suggests an interaction between childhood TE and somatic symptoms as a function of mTBI and PPCS. Childhood trauma may impact the likelihood for mTBI and PPCS through expression of psychological distress as physical symptoms. A greater number of childhood traumatic experiences was also associated with lower levels of interpersonal sensitivity, but only among those with mTBI and PPCS. This may contribute to less socialization given interpersonal sensitivity is necessary to develop healthy relationships. Thus, increased clinical visits which can serve as a means for greater social interactions, may not work as well to fully improve mental health issues due to childhood traumatic experiences. Given a large number of literature has shown ACEs related to poor psychological outcomes during adulthood, particularly among women (11), future studies with a larger sample size are needed to investigate modification by sex in the relationships that were investigated in this study.

Although it was surprising to find a greater number of lifetime traumatic experiences associated with higher levels of mental health (specifically, somatization) among those with mTBI and *no* PPCS (but not with mTBI and PPCS), some potential explanations may help in understanding this result. Individuals with mTBI and PPCS may likely receive more (and variable) treatment for their persistent symptoms which may partially address mental health needs and attenuate mental health symptoms. A greater number of visits may also equate to a greater level of socialization that can improve mood. The length of time managing persistent symptoms may also help gain or develop coping mechanisms to get through the struggles of post-concussion recovery. Additionally, this may also help build a strong social support network known to protect against mental health symptomatology. The presence of lifetime traumatic experiences may also serve as a catalyst to develop mental toughness. This benefit in light of facing difficulties has been coined “stress inoculation” whereby exposure to some adversity can lead to mental toughness, given ample time in between events for recovery (39, 40). Such toughness is thought to be associated with psychological and physiological changes that impact perception of stressful events as being manageable and thus allow for better coping (40).

The findings of this study, though preliminary, provide support for the clinical evaluation of lifetime and childhood traumatic experiences of SMs who have sustained mTBI. This, in turn, may increase the understanding of recovery progression and expectation, which may inform treatment of patients for mTBI. Such assessments may identify individuals at risk for poor recovery, particularly for PPCS. The potential addition of treatment to address lifetime and childhood traumatic experiences may attenuate poor recovery. Additionally, it may improve mental health issues such as somatization and interpersonal sensitivity stemming from childhood traumatic experiences, particularly among those with mTBI and PPCS. Although mental health treatment for trauma is prevalent, challenges still exist in reporting and completion of treatment for mental health issues. As such, a comprehensive assessment of factors including lifetime and childhood trauma that may impact the persistence of symptoms may enhance the recovery trajectory of SMs who present with mTBI.

### 4.1 Limitations

There were a few limitations in this study. First, a cross-sectional approach was utilized and, thus, determination of temporality was not possible, with the exception of the results for ACEs. The study was also predominantly male and non-Hispanic White, and all were SMs. Therefore, the findings may not be generalizable to other populations, and assessment by sex, as suggested by other studies, was not possible. The standard cut-off score of 63 for the BSI dimension and global indices were used to determine clinical relevance (29, 41); however, there were few participants whose score reached above 63. As such, although this study found overall higher scores for BSI dimension and global indices, participants generally did not have clinically meaningful symptomatology. It is still important, however, to demonstrate the contributions of lifetime and childhood trauma on mental health symptoms among SMs with mTBI. A population with higher levels of mental health symptoms may benefit from assessment of lifetime and childhood trauma to prevent exacerbation of mental health symptoms to clinically concerning levels. This study also did not assess specific deployment settings (e.g., combat vs. non-combat) or military occupational specialty which may differ in the level of stress that may impact persistent post-concussive symptoms and/or mental health. This study was also limited in sample size, partly driven by the transient nature of the military community studied, as well as the inclusion of costly investigations such as functional and structural magnetic resonance imaging that was a part of the original protocol. Although this study utilized bootstrapped methods to address the limited sample size, it did not increase the statistical power. Multiple comparisons were also not considered and we could not account for confounders or investigate modifiers given the limited number of participants. A larger sample size is needed to incorporate such statistical approaches and to possibly identify significant relationships that were otherwise found null in this study. Future studies with a larger sample and longitudinal design are needed to verify the significant relationships found in this study and to address the discussed limitations.

### 4.2 Conclusions

In this exploratory study, a greater number of lifetime trauma was found to be associated with PPCS, but elevations of mental health did not explain the relationship. Indeed, a greater number of lifetime trauma might be associated with more somatization, but only among those without PPCS. The effect size between the number of childhood trauma and PPCS was moderate, and a greater number of such events was shown related to greater levels of somatization and lower levels of interpersonal sensitivity exclusively among those with PPCS. Screening for lifetime and childhood trauma during initial evaluations may improve risk assessment, treatment planning, and recovery expectations and outcomes. While this study's results may highlight the need for early identification of at-risk individuals to enhance warfighter readiness and return-to-duty rates, further studies are needed to elucidate the mechanisms of these associations.

## Data Availability

The data analyzed in this study is subject to the following licenses/restrictions: Information and the policies regarding limitations on sharing DoD/DHA data publicly, without an approved Data Sharing Agreement Application (DSAA), can be found at the following website (https://www.health.mil/Military-Health-Topics/Privacy-and-Civil-Liberties/Submit-a-Data-Sharing-Application). The specific DoD Directive (DoDD) that speaks to why we cannot simply share data, even a minimal, de-identified dataset, is DoDD 5400.11 (please refer to additional documentation titled ‘DoDD-5400.11'). In order to access DoD/DHA data, a DSAA must be submitted to the Privacy Office. The appropriate point of contact to initiate the DSAA process can be reached at DHA.DataSharing@health.mil. This DSAA would be between TBICoE and the intended recipient of the data and would need to be requested by the recipient and signatures obtained from all party's authorities. The DSAA would outline the intended use and retention of the data, which will be reviewed by the Privacy Board (estimated review time is between 3–6 months depending on the type of request). A determination will then be made by the Privacy Board based on whether the intended use of the data by the recipient meets the standards of the DoD Privacy Program. Approval of DSAA is subject to Privacy Board review. For further information, questions can be submitted to the Privacy Office at DHA.DataSharing@health.mil.

## Author disclaimer

The views expressed in this manuscript are those of the authors and do not necessarily represent the official policy or position of the Defense Health Agency, Department of War, or any other U.S. Government agency. This work was prepared under Contract HT0014-22-C-0016 with DHA Contracting Office (NM-CD) HT0014 and, therefore, is defined as U.S. Government work under Title 17 U.S.C.§101. Per Title 17 U.S.C.§105, copyright protection is not available for any work of the U.S. Government. For more information, please contact dha.TBICOEinfo@health.mil.

## References

[1] Traumatic Brain Injury Center of Excellence. DoD Numbers for TBI Worldwide. Falls Church, VA: Defense Health Agency (2025). Available online at: https://health.mil/TBINumbers (Accessed Sepember 18, 2025).

[2] Eme R. Neurobehavioral outcomes of mild traumatic brain injury: a mini review. Brain Sci. (2017) 7:46. doi: 10.3390/brainsci7050046 28441336 PMC5447928

[3] Howlett JR Nelson LD Stein M. Mental health consequences of traumatic brain injury. Biol Psychiatry. (2023) 91:413–20. doi: 10.1016/j.biopsych.2021.09.024 PMC8849136 34893317

[4] Sheldrake E Al-Hakeem H Lam B Goldstein BI Wheeler AL Burke M . Mental health outcomes across the lifespan in individuals with persistent post-concussion symptoms: a scoping review. Front Neurol. (2022) 13:850590. doi: 10.3389/fneur.2022.850590 35481264 PMC9035995

[5] Reid MW Velez CS. Discriminating military and civilian traumatic brain injuries. Mol Cell Neurosci. (2015) 66:123–8. doi: 10.1016/j.mcn.2015.03.014 25827093

[6] Rigney G Jo J Williams K Terry DP Zuckerman SL. Parental factors associated with recovery after mild traumatic brain injury: a systematic review. J Neurotrauma. (2023) 40:2015. doi: 10.1089/neu.2023.0015 37212287

[7] Walker EA Glefand A Katon WJ Koss MP Korff MV Bernstein D . Adult health status of women with histories of childhood abuse and neglect. Am J Med. (1999) 107:332–9. doi: 10.1016/S0002-9343(99)00235-1 10527034

[8] MacMillan HL Fleming JE Streiner DL Lin E Boyle MH Jamieson E . Childhood abuse and lifetime psychopathology in a community sample. Am J Psychiatry. (2001) 158:1878–83. doi: 10.1176/appi.ajp.158.11.1878 11691695

[9] Felitti VJ Anda RF Nordenberg D Williamson DF Spitz AM Edwards VJ . Relationship of childhood abuse and household dysfunction to many of the leading causes of death in adults: the adverse childhood experiences (ACE) study. Am J Prev Med. (2019) 56:774–86. doi: 10.1016/j.amepre.2019.04.001 31104722

[10] Lynch KS Lachman ME. The effects of lifetime trauma expsore on cognitive functioning in midlife. J Trauma Stress. (2020) 33:773–82. doi: 10.1002/jts.22522 32516491 PMC7572703

[11] Remigio-Baker RA Hayes DK Reyes-Salvail F. Adverse childhood events and current depressive symptoms among women in Hawaii: 2010 BRFSS, Hawaii. Matern Child Health J. (2014) 18:2300–8. doi: 10.1007/s10995-013-1374-y 24178156

[12] VanMeter F Nivison MD Englund MM Carlson EA Roisman GI. Childhood abuse and neglect and self-reported symptoms of psychopathology through midlife. Dev Psychol. (2021) 57:824–36. doi: 10.1037/dev0001169 34166025 PMC8284929

[13] Yin R Yang Y Tang L Chang Y Zhang F. Childhood abuse and association with adult depressive symptoms among people with cardiovascular disease. Front Public Health. (2023) 2:1179384. doi: 10.3389/fpubh.2023.1179384 PMC10273208 37333526

[14] Edwards VJ Holden GW Felitti VJ Anda RF. Relationship between multiple forms of childhood maltreatment and adult mental health in community respondents: results from the adverse childhood experiences study. Am J Psychiatry. (2003) 160:1453–60. doi: 10.1176/appi.ajp.160.8.1453 12900308

[15] Copeland WE Shanahan L Hinesley J Chan RF Aberg KA Fairbank JA . Association of childhood trauma exposure with adult psychiatric disorders and functional outcomes. JAMA Netw Open. (2018) 1:e184493. doi: 10.1001/jamanetworkopen.2018.4493 30646356 PMC6324370

[16] Dobernecker J Spyridou A Elbert T Schauer M Garthus-Niegel S Ruf-Leuschner M . Cumulative trauma predicts hair cortisol concerntrations and symptoms of depression and anxiety in pregnant women—an investigation of community samples from Greece, Spain and Peru. Sci Rep. (2023) 13:1434. doi: 10.1038/s41598-023-28151-9 36697477 PMC9876917

[17] Sacchi L Merzhvynska M Augsburger M. Effects of cumulative trauma load on long-term trajectories of life satisfaction and health in a population-based study. BMC Public Health. (2020) 20:1612. doi: 10.1186/s12889-020-09663-9 33109171 PMC7590721

[18] Remigio-Baker RA Hayes DK Reyes-Salvail F. Adverse childhood events are related to the prevalence of asthma and chronic obstructive pulmonary disorder among women in Hawaii. Lung. (2015) 193:885–91. doi: 10.1007/s00408-015-9777-8 26267594 PMC4654662

[19] Kim Y Lee H Park A. Patterns of adverse childhood experiences and depressive symptoms: self-esteem as a mediating mechanism. Soc Psychiatry Psychiatr Epidemiol. (2022) 57:331–41. doi: 10.1007/s00127-021-02129-2 34191037 PMC8243305

[20] Weil ZM White B Whitehead B Karelina K. The role of the stress system in recovery after traumatic brain injury: a tribute to Bruce S. McEwen. Neurobiol Stress. (2022) 19:100467. doi: 10.1016/j.ynstr.2022.100467 35720260 PMC9201063

[21] Cancellierre C Mohammed RJ. Brain drain: psychosocial factors influence recovery following mild traumatic brain injury-−3 recommendations for clinicians assessing psychosocial factors. J Orthop Sports Phys Ther. (2019) 49:842–4. doi: 10.2519/jospt.2019.8849 31154953

[22] Department of Veterans Affairs/Department of Defense. VA/DoD Clinical Practice Guideline for the Management of Concussion-Mild Traumatic Brain Injury. Washington, DC: Department of Veterans Affairs/Department of Defense (2016). Available online at: https://www.va.gov/covidtraining/docs/mTBICPGFullCPG50821816.pdf (Accessed October 20, 2025)

[23] Weathers FW Blake DD Schnurr PP Kaloupek DG Marx BP Keane TM. The Clinician-Administered PTSD Scale for DSM-5 (CAPS-5)—Past Week 2015 (2015). Available online at: https://www.ptsd.va.gov/professional/assessment/adjust-int/caps.asp (Accessed April 16, 2025)

[24] Weathers FW Bovin MJ Lee DJ Sloan DM Schnurr PP Kaloupek DG . The clinician-administered PTSD scale for DSM-5 (CAPS-5): development and initial psychometric evaluation in military veterans. Psychol Assess. (2017) 30:383–95. doi: 10.1037/pas0000486 28493729 PMC5805662

[25] Zuromski KL Ustun B Hwang I Keane TM Marx BP Stein MB . Developing an optimal short-form of the PTSD checklist for DSM-5 (PCL-5). Depress Anxiety. (2019) 36:790–800. doi: 10.1002/da.22942 31356709 PMC6736721

[26] Blevins CA Weathers FW Davis MT Witte TK Domino JL. The post-traumatic stress disorder checklist for DSM-5 (PCL-5): development and initial psychometric evaluation. J Trauma Stress. (2015) 28:489–98. doi: 10.1002/jts.22059 26606250

[27] Derogatis LR. Brief Symptom Inventory (BSI). Minneapolis, MN: National Computer Systems, Inc. (1975). Available online at: https://hazards.colorado.edu/nhcdata/chernobyl/ChData/ScalesInstruments/Scales%20and%20Indices/Scale%20Construction%20Instructions/BSI.pdf (Accessed April 16, 2025)

[28] Conoley JC Kramer JJ. The Tenth Mental Measurements Yearbook. Lincoln, NE: University of Nebraska Press (1989). p. 111–3.

[29] Derogatis LR. BSI Brief Symptom Inventory. Administration, Scoring, and Procedures Manual. 4th ed. Minneapolis, MN: National Computer Systems (1993).

[30] Carlson EB Smith SR Palmieri PA Dalenberg CJ Ruzek JI Kimerling R . Development and validation of a brief self-report measure of trauma exposure: the trauma history screen. Psychol Assess. (2011) 23:463–77. doi: 10.1037/a0022294 21517189 PMC3115408

[31] Mei X Li JJ Li Z-S Huang S Li L-L Huang Y-H . Psychometric evaluation of an adverse childhood experience (ACEs) measurement tool: an equitable assessment or reinforcing bias? Health Justice. (2022) 10:34. doi: 10.1186/s40352-022-00198-2 36445502 PMC9706892

[32] Asheh AM Courchesne-Krak N Kepner W Marienfeld C. Adverse childhood experiences are associated with history of overdose among patients presenting for outpatient addiciton care. J Addict Med. (2023) 17:333–8. doi: 10.1097/ADM.0000000000001126 37267182 PMC10241414

[33] Plouffe RA Easterbrook B Liu A McKinnon MC Richardson JD Nazarov A. Psychometric evaluation of the moral injury events scale in two Canadian armed forces samples. Assessment. (2023) 30:111–23. doi: 10.1177/10731911211044198 34515535

[34] Schauss E Zettler H Patel M Hawes K Dixon P Bartelli D. Exploring the test-retest differences of self-reported adverse childhood experiences among adolescents in residential treatment. J Fam Trauma Child Custody Child Dev. (2021) 18:263–78. doi: 10.1080/26904586.2021.1918037

[35] Meshberg-Cohen S Cook JM Fischer IC Pietrzak RH. Mild traumatic brain injury in U. S military veterans: results from the National Health and Resilience in Veterans Study. Psychiatry. (2024) 87:314–28. doi: 10.1080/00332747.2024.2392226 39186319

[36] McKay MT Cannon M Chambers D Conroy RM Coughlan H Dodd P . Childhood trauma and adult mental disorder: a systematic review and meta-analysis of longitudinal cohort studies. Acta Psychatr Scand. (2021) 143:189–205. doi: 10.1111/acps.13268 33315268

[37] Kessler RC Aguilar-Gaxiola S Alonso J Benjet C Bromet EJ Cardoso G . Trauma and PTSD in the WHO World Mental Health Surveys. Eur J Psychotraumatol. (2017) 8:1353383. doi: 10.1080/20008198.2017.1353383 29075426 PMC5632781

[38] Benjet C Bromet E Karam EG Kessler RC McLaughlin KA Ruscio AM . The epidemiology of traumatic event exposure worldwide: results from the World Mental Health Survey Consortium. Psychol Med. (2015) 46:327–43. doi: 10.1017/S0033291715001981 26511595 PMC4869975

[39] Meichenbaum D. “Principles and practices of stress management”. In:Woolfolk RL Lehrer PM, editors. Stress Inoculation Training: A Twenty Year Update. 2nd ed. New York, NY: Guilford Press (1993). p. 373–406.

[40] Dienstbier RA. Arousal and physiological toughness: implications for mental and physical health. Psychol Rev. (1989) 96:84–100. doi: 10.1037/0033-295X.96.1.84 2538855

[41] Hart T Benn EKT Bagiella E Arenth P Dikmen S Hesdorffer DC . Early trajectory of psychiatric symptoms after traumatic brain injury: relationship to patient and injury characteristics. J Neurotrauma. (2014) 31:610–7. doi: 10.1089/neu.2013.3041 24237113 PMC3961785

